# Unexplained High Prevalence of ESBL-*Escherichia coli* Among Cattle and Pigs in Peru

**DOI:** 10.3390/antibiotics14090867

**Published:** 2025-08-28

**Authors:** Marília Salgado-Caxito, Daphne Léon, Olga Bardales, Luis M. Jara, Patricia Medrano, Clara Murga, Veronica Pérez, Brenda Aylas-Jurado, Roberto Su-Tello, Juana Najarro, Elías Salvador-Tasayco, Jonas Farrugia-Audri, Carlos Shiva, Julio A. Benavides

**Affiliations:** 1 UMR MIVEGEC, IRD, CNRS, University of Montpellier, 34394 Montpellier, France; mariliasalgadocaxito@gmail.com (M.S.-C.); daphne.leon@upch.pe (D.L.); farrugiajonas@gmail.com (J.F.-A.); 2 Facultad de Medicina Veterinaria y Zootecnia, Universidad Peruana Cayetano Heredia, Lima 15102, Peru; luis.jara.s@upch.pe (L.M.J.); patricia.medrano.r@upch.pe (P.M.); clara.murga@upch.pe (C.M.); veronica.castro@upch.pe (V.P.); brenda.aylas.j@gmail.com (B.A.-J.); roberto.su.t@upch.pe (R.S.-T.); juana.najarro.p@upch.pe (J.N.); 3 Facultad de Educación, Universidad Peruana Cayetano Heredia, Lima 15102, Peru; olga.bardales.m@upch.pe; 4 Emerge, Unidad de Investigación en Enfermedades Emergentes y Cambio Climático, Facultad de Salud Pública y Administración, Universidad Peruana Cayetano Heredia, Lima 15102, Peru; 5 Facultad de Medicina Veterinaria y Zootecnia, Universidad Nacional San Luis Gonzaga, Ica 11002, Peru; elias.salvador@unica.edu.pe; 6 One Health Institute and Doctorado en Medicina de la Conservación, Faculty of Life Sciences, Universidad Andres Bello, Santiago 8320000, Chile

**Keywords:** Extended-Spectrum Beta-Lactamases, dairy cows, pigs, poultry, Latin America, farmer, questionnaires

## Abstract

**Background/Objectives**: Extended-Spectrum Beta-Lactamase-producing *Escherichia coli* (ESBL-*E. coli*) are widely circulating in livestock of low- and middle-income countries. However, the drivers of their circulation remain largely unknown. Small-scale farms in Peru exhibit an unusually high prevalence of fecal carriage of ESBL-*E. coli* in their livestock. The objective of this study was to compare the prevalence of ESBL-*E. coli* fecal carriage in dairy cows, pigs, and poultry in the Lima and Ica regions of Peru and to identify the drivers associated with the observed prevalence at the farm level. **Methods**: We compared the prevalence of fecal carriage of ESBL-*E. coli* isolated from dairy cattle (N = 244 animals; 25 farms), pigs (N = 261; 25), and laying hens (N = 255; 10). We also administrated questionnaires to 59 farmers regarding their socioeconomic status, husbandry practices, animal diseases, and antibiotic use. **Results**: All but one of the 60 farms sampled had at least one animal carrying ESBL-*E. coli*. A statistically higher prevalence of ESBL-*E. coli* was estimated in dairy cows (75%) and pigs (61%) from Lima compared to laying hens from Ica (34%). Our statistical analyses (Poisson generalized linear models) using two variable selection approaches revealed that the prevalence of ESBL-*E. coli* was lower in farms raising laying hens, when farmers oversaw both animal husbandry and healthcare, and in farms with a lower number of gastrointestinal outbreaks in the last semester. Socio-economic features of farmers and self-reporting antibiotic use varied across farms (i.e., highest antibiotic use over the last semester was reported among pig farmers (96%), followed by laying hen (70%) and dairy cattle farmers (50%)), but these factors were not associated with the prevalence of ESBL-*E. coli*. **Conclusions**: Despite a relatively low number of farms sampled, our findings suggest that the widespread circulation of ESBL-*E. coli* among livestock in Peru could be mainly associated with unknown species-specific drivers, independently of the socioeconomic status of farmers and antibiotic use. Therefore, our study calls for future research to identify the specific drivers promoting the high prevalence of ESBL-*E. coli* among cattle and pigs in Peru.

## 1. Introduction

Antimicrobial resistance (AMR) is a major concern for global public health that can be exacerbated in low- and middle-income countries (LMICs) [1]. In fact, bacterial infections are a leading cause of death in these countries [2], given fewer antibiotic options for treatment and limited access to healthcare compared to high-income countries [3,4]. Extended-Spectrum Beta-Lactamase-producing *Escherichia coli* (ESBL-*E. coli*) are the leading cause of human deaths attributed to AMR worldwide [1], and their prevalence has also risen in livestock in recent decades [5], with still unknown consequences in terms of global and local economic impact. For example, ESBL-*E. coli* have been reported in both healthy and sick livestock in different LMIC farm settings [5,6,7,8]. Therefore, understanding the drivers explaining the rise of ESBL-*E. coli* in the livestock sector of LMICs is essential to reduce the burden of AMR in both humans and animals.

The increasing antimicrobial use (AMU) and misuse in the livestock sector is a major contributor to the selection and rise of ESBL-*E. coli* among these animals from LMICs [9,10]. However, several context-specific farm practices, including sanitation, hygiene, and disease incidence, can also influence the circulation of ESBL-*E. coli* among small-scale farms, which represent the majority of the livestock sector in LMICs [11,12,13]. For example, a lack of water quality control, large people flow (e.g., visiting farmers), and poor farm biosecurity can be associated with bacterial infections among farms [14,15,16], increasing AMU on livestock and thus the selection of ESBL-*E. coli* [17]. Furthermore, the socio-cultural and economic characteristics of farmers can influence their animal healthcare and sanitation practices, since historical social and educational gaps in LMICs are reflected in the farming sector. For example, farmers lacking access to technology and technical knowledge for animal care often rely on practices based on validated traditional knowledge transmitted generationally as true and effective [18,19,20]. In fact, our previous studies suggested that a lack of formal education, limited access to veterinary services, and reduced financial resources are linked to the lack of adoption of biosecurity behaviors and antibiotic misuse in livestock [6,21]. However, the context-specific drivers associated with a high occurrence of ESBL-*E. coli* among livestock remain poorly understood. Identifying such drivers is crucial for the planning of efficient interventions aiming to reduce ESBL-*E. coli* circulation in rural environments of LMICs.

Peru is a middle-income country struggling with AMR in the livestock sector, given widespread misuse of antibiotics, along with poor and farmers’ traditional knowledge on antibiotics [21,22,23]. In particular, an increase of 160% in livestock AMU was predicted between 2015 and 2030 [9]. The poultry sector is the largest in the country, with 191.7 million chickens contributing to 24% of the national Gross Livestock Production Value (GLVP) in 2023 (USD ~ 2.65 billion) [24]. Although smaller in number of animals, cattle and pig farming are the second and third largest contributors to the Peruvian livestock economy, with 6.8 million beef and dairy cattle (8.7% of the GLVP) and 3.5 million pigs (2.4% of the GLVP). Small-scale farms represent the large majority (82%) of Peru’s livestock sector and are mainly run by low-income farmers [25,26,27]. The Peruvian National Plan to Combat AMR includes a One Health approach to reduce the circulation of AMR [28]. The objectives of this national plan include identifying the knowledge, attitudes, and practices towards AMR, implementing AMR surveillance and research, and reducing the incidence of bacterial infections with appropriate sanitary, hygiene, and prevention measures. However, the current lack of AMR surveillance in the Peruvian livestock sector, as well as limited information on farm features and farmer practices related to the treatment of bacterial infections, precludes the identification of the main drivers of AMR that can then be hindered with appropriate targeted interventions. Therefore, this study aimed to combine laboratory screening of AMR in livestock with farmer questionnaires to identify the main factors associated with AMR across farms raising different livestock species in the coastal regions of Peru.

In Peru, the coastal regions of Lima and Ica concentrate a large part of the milk, pig meat, and egg production, contributing together with over 25% of the GLPV in 2020 [24]. However, these two regions have reported an unusually high circulation of ESBL-*E. coli* among their animals compared to other countries of Latin America, with an estimated prevalence of ESBL-*E. coli* fecal carriage ranging from 48% in cows and pigs of Lima to 72% in chickens of Ica [6,23,29]. These prevalence estimates are much higher than those reported in similar small-scale livestock farms of neighboring countries, ranging from 3% in multiple livestock species of Chile [7] to 18% in poultry of Brazil [30,31]. However, the drivers associated with this high ESBL-*E. coli* prevalence in livestock of Lima and Ica have not been identified. The misuse of antibiotics in these regions has been suspected as a key factor promoting the selection of these bacteria [21,22]. However, to our knowledge, no study has tested the association between the ESBL-*E. coli* fecal carriage in Peruvian livestock and antibiotic use and other factors, nor have they compared ESBL-*E. coli* prevalence between livestock species.

Practices associated with raising a particular livestock species (e.g., cattle, pigs, or poultry) can affect the likelihood of ESBL-*E. coli* circulation [32]. For example, studies in high-income countries have suggested a higher likelihood of antibiotic-resistant bacteria, including cefotaxime-resistant *E. coli*, among pigs compared to cattle, poultry, and sheep under similar husbandry conditions [33,34,35]. However, comparisons in the prevalence of ESBL-*E. coli* across different livestock species and the drivers behind potential differences associated with the livestock species of LMICs have received little attention [36,37]. The few published examples on this comparison include a higher ESBL-*E. coli* prevalence in poultry compared to other species (i.e., pigs, cattle, small ruminants, and rabbits) in relatively low-income regions of Guadeloupe [38], the Reunion Island [39], and Malaysia [40]. In contrast, ESBL-*E. coli* prevalence was not different in feces collected from pigs and cattle of Argentina [41]. The multifactorial nature of ESBL-*E. coli* circulation among the livestock of LMICs underscores the need for cross-sectional studies aiming to identify context-specific drivers of AMR such as ESBL-*E. coli,* which can then be used to inform the development of tailored interventions targeting small-scale farming systems.

Thus, this study aimed to (i) compare the prevalence of ESBL-*E. coli* fecal carriage among dairy cows, pigs, and laying hens from small farms of the highly productive regions of Lima and Ica in Peru, and (ii) identify the socioeconomic factors and husbandry practices associated with ESBL-*E. coli* prevalence at the farm-level.

## 2. Results

### 2.1. Prevalence of ESBL-E. coli Fecal Carriage by Livestock Species

**Figure 1:**
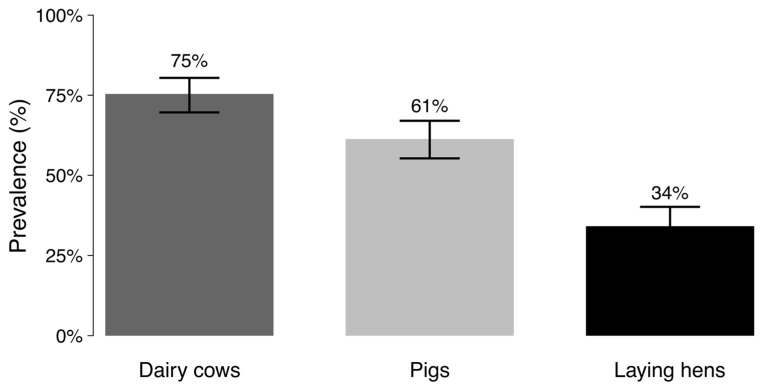
Prevalence of ESBL-*E. coli* in dairy cows and pigs from small-scale farms in Lima and laying hens from Ica, Peru. Lines correspond to the estimated 95% confidence intervals calculated in R using the Agresti–Coull method [42].

Fifty-nine out of the sixty farms sampled (98% prevalence) had at least one animal carrying ESBL-*E. coli*. ESBL-*E. coli* were isolated from feces in 432 out of 760 sampled animals (prevalence of 56.7% [95% CI = 53.2–60.2%]). The prevalence of ESBL-*E. coli* was higher in dairy cows (75.4% [95% CI = 69.6–80.4%]) compared to pigs (61.3% [95% CI = 55.3–67%], Chi-square test with the Bonferroni correction, *p* < 0.05) and laying hens (34.1% [95% CI = 28.6–40.1%]). Prevalence of ESBL-*E. coli* was also higher in pigs compared to laying hens (Chi-square test with the Bonferroni correction, *p* < 0.05) (Figure 1). The large majority (96%, 75 out of 78) of a random selection of these ESBL-*E. coli* isolates harbored the ESBL gene *bla*_CTX-M_, either alone or in combination with the genes *bla*_TEM_ (32%, 25/78) and *bla*_SHV_ (3%, 2/78). While all livestock species showed a predominant carriage of *bla*_CTX-M_, *bla*_TEM_ was only observed in laying hens and pigs, and *bla*_SHV_ in dairy cows and pigs.

### 2.2. Farmers’ Socioeconomic Characteristics

We interviewed 59 farmers regarding their socioeconomic status and the main activities carried out daily on their farms. Most farmers (34%, 20/59) were men under 50 years old with at least an elementary education (Table 1). The absence of formal education was exclusively reported among women over 51 years old. Farmers’ daily activities primarily involved animal husbandry tasks, including animal feeding, milking, or collecting eggs, inspecting animals(e.g., identifying sick animals), and performing farm biosecurity measures (e.g., cleaning and disinfecting animal pens). They were also responsible for deciding on necessary actions for sick animals, such as contacting a veterinarian or initiating antibiotic treatments. However, only 30% (18/59) personally handled healthcare-related tasks like examining sick animals and administering treatments.

### 2.3. Differences Among Livestock Species

We gathered information on each farm’s characteristics and husbandry practices, the occurrence and management of diseases in the herd (e.g., details of animal AMU over the last semester/year), and biosecurity measures across the surveyed dairy cattle, pig, and laying hen farms. Herd sized varied significantly by livestock species: dairy cattle farms (n = 24) had a median of 18 animals (mean = 68, standard deviation (sd) = 213, range = 9–1065), pig farms (n = 25) had a median of 80 animals (mean = 101, sd = 76, range = 12–300), and laying hen farms (n = 10) had a median of 12,585 animals (mean = 18,860, sd = 19,154, range = 850–68,000). Dairy cattle farms primarily used natural streams as water sources (54%, 13/24), while pig farms relied almost exclusively on wells (96%, 24/25), and all 10 surveyed laying hen farms used potable water. Feeding practices also differed significantly across livestock species. For example, all dairy cattle and laying hen farmers provided commercial and balanced feed to their animals, whereas 84% (21/25) of pig farmers used human food waste, either alone or mixed with commercial feed.

**Figure 2:**
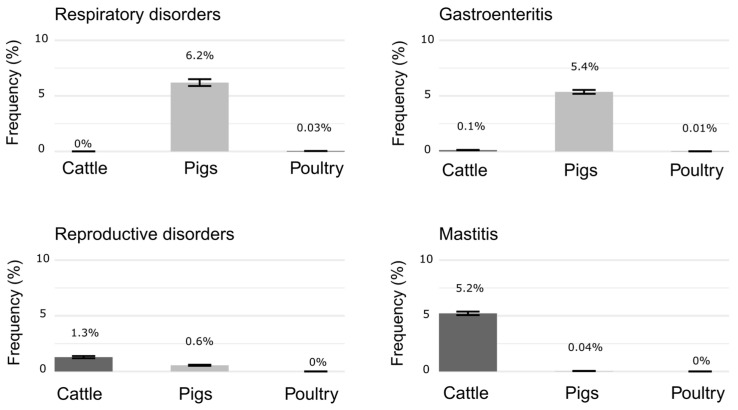
Frequency and standard deviation (sd) of respiratory, gastroenteric, reproductive, and mastitis diseases affecting livestock by animal species, estimated as the sum of reported occurrences in the last semester by farmers, divided by the total number of animals on their farms.

Differences across livestock species were observed regarding diseases affecting the herds (Figure 2). Mastitis was the most reported disease in dairy cows, while respiratory diseases were more often reported in pigs and in laying hens (but at a lower rate). Sick animal management also differed. Only 42% (10/24) of dairy cattle farmers separated sick from asymptomatic animals, compared to 84% (21/25) of pig and 80% (8/10) of laying hen farmers. Most pig farmers (80%, 20/25) reported relying on public veterinary services (SENASA) as their only access to veterinary care, while dairy cattle (88%, 21/24) and laying hen (90%, 9/10) farmers mentioned using both public and private services.

**Figure 3:**
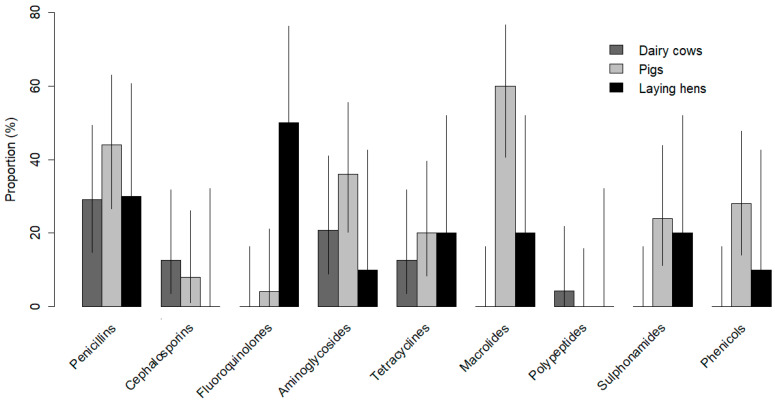
Proportion of farmers raising dairy cattle, pigs, and laying hens in Peru (Lima and Ica regions) reporting the use of at least one of nine antibiotic categories to treat respiratory, gastrointestinal, reproductive, or mastitis infections in their livestock. Black lines indicate 95% confidence intervals of the estimated proportions using the Agresti–Coull method of the *binom.confint* function in R 4.2.1.

Overall, AMU was mainly reported by pig farmers (96% in the past year and semester), closely followed by laying hen farmers (90% in the past year, and 70% in the last semester), and to a lower extent by dairy cattle farmers (54% in the past year, and 50% in the last semester). The main antibiotic categories used for livestock treatments in the last year varied between species (Figure 3). Dairy cattle farmers mainly reported using penicillins, pig farmers mainly reported using macrolides, and laying hen farmers mainly reported using fluoroquinolones. These antibiotics were used to treat the most reported diseases in the herds. However, the antibiotic choice changed when applied to infrequent diseases among dairy cattle and laying hen farmers. For instance, dairy farmers tended to treat reproductive disorders with cephalosporins and laying hen farmers mostly used penicillins (specifically amoxicillin) for gastroenteritis treatments.

**Figure 4:**
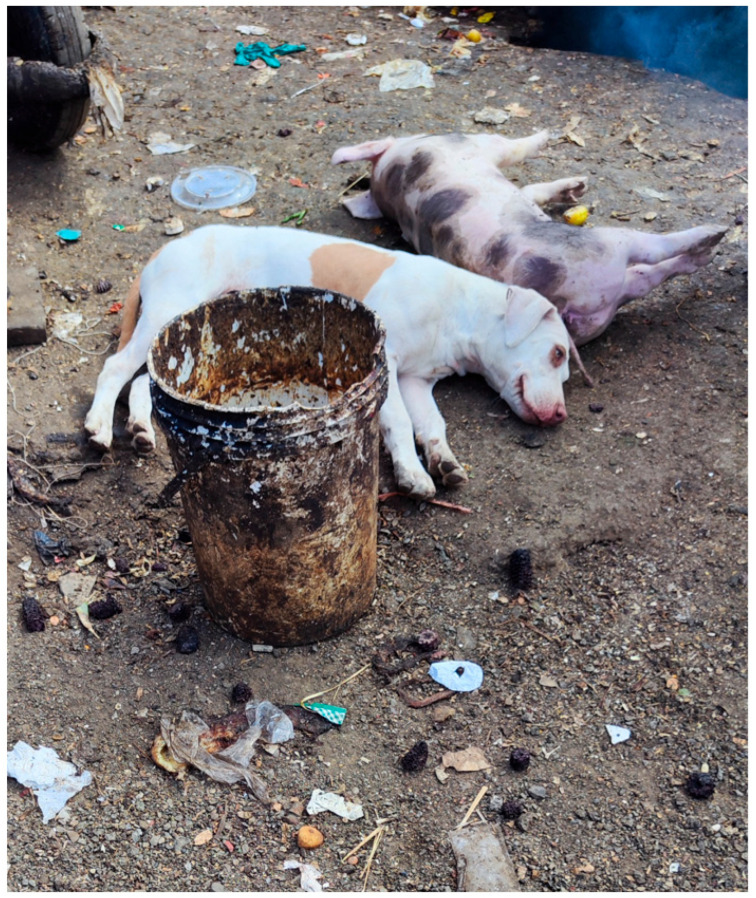
Visible waste scattered across animal pens and livestock–dog contact at a sampled pig farm in Lima, Peru. Photo credit: Clara Murga.

Regarding biosecurity practices, rodent presence was common across all farms but more prevalent in pig (80%, 20/25) and laying hen farms (80%, 8/10). However, laying hen farms reported higher rodent control (80%, 8/10) compared to pig (40%, 10/25) and dairy cattle farms (8%, 2/24). Wastewater treatment (i.e., locally or using public services) was more common in pig (80%, 20/25) and laying hen farms (60%, 6/10), while most dairy cattle farms (58%, 14/24) discharged untreated wastewater into the environment. Additionally, 17% (4/24) of dairy cattle and 10% (one farm) of laying hen farms used livestock wastewater for agriculture. Cleaning and disinfection practices were reported on all but one farm. Daily cleaning was performed in 75% (18/24) of dairy cattle farms and 92% (23/25) of pig farms, and monthly cleaning was reported in 50% (5/10) of laying hen farms. Disinfection was mostly conducted daily in dairy cattle farms (67%, 16/24), monthly in pig farms (80%, 20/25), and weekly in poultry farms (70%, 7/10). However, field observations indicated inconsistencies, with visible waste in many animal pens at the time of animal sampling (Figure 4). Boot/wheels baths were rare, used in only 8% of surveyed farms (5 out of 59, including one dairy cattle and four pig farms). Livestock–dog contact was common across all farms (Figure 4), including in 83% (20/24) of dairy cattle, 80% (8/10) of laying hen, and 52% (13/25) of pig farms.

### 2.4. Statistical Analyses to Identify Factors Correlated to ESBL-E. coli Prevalence

**Figure 5:**
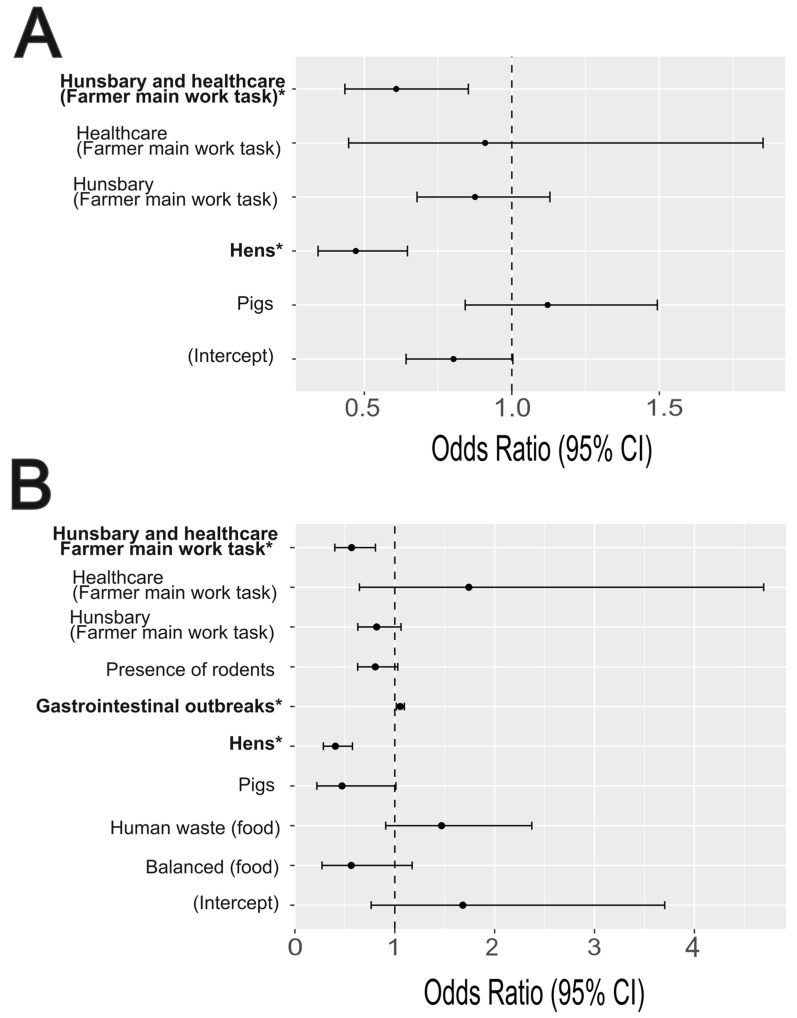
Forest plots for the Odds Ratios (ORs) and their 95% confidence intervals (solid lines) for factors associated with ESBL-*E. coli* rates in livestock of small-scale farms of Peru obtained from the selected Poisson regression model. (**A**) Model obtained with the LASSO procedure to select variables; (**B**) model obtained with the AIC selection over a model including 14 selected variables from the 6 categories of the questionnaire. The following values were used in the model as a reference to estimate the effect of other categories: ‘Administrative’ (farmer’s main work tasks) and ‘Cattle’ (animal species). The vertical dash line represents the null value for the odds ratio (OR = 1). Significant variables (*p* < 0.05) are marked with an asterisk (*). Plot created using the *plot_model* function of the *sjPlot* package in R 4.2.1.

Using the LASSO selection method, the obtained Poisson GLM included ‘animal species’ and ‘farmer main work tasks’, with an offset for the total number of animals tested (Figure 5). This model showed a good fit to the data, with a residual deviance of 58.4 on 44 degrees of freedom and an AIC of 256.96. The residual plots showed no significant overdispersion (*p* = 0.86), outliers (*p* = 1), nor deviation from uniformity (KS test *p* = 0.50). In this model, the prevalence of ESBL-*E. coli* was significantly lower in laying hens compared with the reference species (cattle) (Poisson GLM, β = −0.75, OR = 0.47 [0.34–0.65], *p* < 0.001). Farmers in charge of both animal healthcare (examining and administering treatments to sick animals) and husbandry tasks were also associated with half the prevalence of ESBL-*E. coli* carriage compared to farmers responsible only for administrative activities (Poisson GLM, β = −0.5, OR = 0.61 [0.43–0.85], *p* < 0.01). Despite differences between livestock species regarding disease occurrence and AMU patterns, none were selected by the LASSO procedure for inclusion in the final model.

The model used in the AIC selection process included the following 13 variables: use of antibiotics or not in animals over the last year, number of gastroenteritis outbreaks in the last semester, number of reproductive disorders in the last semester, frequency of farm disinfection, contact or not between livestock and companion animals, animal species raised, presence/absence of rodents, type of food provided to animals, isolation or not of animals when sick, farmer’s age, gender, education level, and main type of task on the farm. The AIC selection retained a model with 5 variables, including the number of gastroenteritis outbreaks in the last semester, animal species kept, presence/absence of rodents, type of food given, and the farmer’s main type of task. This model also showed a good fit to the data, with no significant overdispersion (*p* = 0.64), outliers (*p* = 1), nor deviation from uniformity (KS test *p* = 0.33). Similar to the LASSO procedure, this model showed a lower prevalence of ESBL-*E. coli* among laying hen farms compare to cattle (Poisson GLM, β = −0.90, OR = 0.41 [0.28–0.58], *p* < 0.001) and a lower prevalence in farms where farmers were in charge of both animal healthcare and husbandry tasks (Poisson GLM, β = −0.57, OR = 0.57 [0.40–0.81], *p* < 0.01) (Figure 5B). In addition to the LASSO procedure, this model also detected a significant positive correlation between the number of outbreaks of gastroenteritis (mainly affecting pigs) and the prevalence of ESBL-*E. coli* carriage (Poisson GLM, β = 0.05, OR = 1.68 [0.76–3.70], *p* < 0.01).

## 3. Discussion

Despite the high prevalence of ESBL-*E. coli* fecal carriage reported in livestock in Peru, the drivers promoting their circulation remain unclear. Our study revealed that almost all farms (98%) had an animal carrying ESBL-*E. coli* and estimated a high ESBL-*E. coli* prevalence of 57% in livestock species from low-income farms in Lima and Ica, despite self-reported low levels of beta-lactam use. Our statistical analyses correlating ESBL-*E. coli* farm-level prevalence with questionnaire responses revealed that ‘livestock species’, ‘farmers’ main work tasks’ and the ‘number of gastrointestinal outbreaks’ were the only factors associated with ESBL-*E. coli*. In contrast, despite variations in farmers’ socio-economic status and self-reported antibiotic use, these variables were not statistically correlated with ESBL-*E. coli* prevalence. The significant differences in ESBL-*E. coli* prevalence between livestock species suggest that unknown species-specific factors may be associated with the circulation of ESBL-*E. coli* in this study area.

The estimated high prevalence of ESBL-*E. coli* in livestock from Lima and Ica (57%) in this study was relatively similar to the one estimated by our previous study conducted six years earlier in the same region among small-scale farmers who mostly kept a small but diverse number of livestock, including cattle, pigs and poultry (48% in 2017; [6]). This sustained high prevalence, likely among the highest in the world, is in line with projections of the global rise in ESBL-*E. coli* prevalence in livestock over the last 10 years [5,43,44]. Both methods used in our statistical analyses found that when the same person handled both animal healthcare and husbandry, the likelihood of ESBL-*E. coli* carriage on that farm was reduced by half. Although further research is needed to determine the specific reason for this association, we propose that farmers managing both tasks may be more capable of reducing the unnecessary use of antibiotics and, consequently, ESBL-*E. coli* selection. In fact, a farmer’s knowledge of the farm can facilitate the implementation of preventive measures to avoid bacterial infections, such as early detection of illness, as well as better monitoring of antibiotic treatment. Furthermore, our AIC-based less conservative statistical analysis also associated a higher number of gastroenteritis outbreaks on a farm with a higher prevalence of ESBL-*E. coli* fecal carriage, which is in line with several studies showing that a higher disease incidence can trigger higher antibiotic use [45,46,47], which can in turn select for antibiotic resistance.

Besides a difference in ESBL-*E. coli* prevalence between the livestock species raised, other common drivers such as antibiotic use or socio-economic features of farmers (that can in turn influence antibiotic use and farming practices) were not significantly associated with ESBL-*E. coli* prevalence. Therefore, our study cannot advise on a particular targeted group to reduce ESBL-*E. coli* burden based on farm and farmer features, except for prioritizing cattle and pig farms. The lack of statistical association between several studied variables reported in questionnaires may be due to low statistical power, given that only 60 farms participated in this study and a relatively low number of animals were sampled per farm to estimate farm-level prevalence. Alternatively, the general features of small-scale farming in this area (e.g., overall poor sanitary conditions) could result in such a high circulation of ESBL-*E. coli* (i.e., all but one farm had an animal infected), making the relative influence of other specific drivers more difficult to detect. Furthermore, most of the variables studied, such as disease occurrence and antibiotic use, depend on the specific livestock species raised (e.g., mastitis infections affected mostly cattle and gastroenteritis mostly pigs), and are therefore already partially included in the effect of the variable ‘livestock species’.

Dairy cows from the studied farms exhibited the highest prevalence of ESBL-*E. coli* (75%). Although our model did not reveal any major statistically significant associations, certain dairy management practices detected through our questionnaires may be contributing to the emergence and spread of these resistant strains, such as inadequate farm waste management leading to bacterial diseases and the need for treatment with antibiotics, which should be further explored in future research. Dairy cattle in this study were primarily affected by mastitis and were mostly treated with penicillins, with a small proportion of farmers using third-generation cephalosporins. In contrast, our previous study in the same region showed that sulphonamides were the main antibiotics used for this type of infection [21], suggesting a potential shift in AMU in this area. *E. coli*-induced mastitis is a major cause of productive loss in dairy cattle worldwide [48]. However, the treatment—generally with fluoroquinolones or broad-spectrum cephalosporins—is recommended only in critical cases, as previous research suggests that mild/moderate *E. coli* infections are self-limiting [48,49,50]. Although we did not assess the severity of reported infections in our study, the empirical use of penicillins suggests inappropriate AMU on the studied farms. Considering *E. coli* as the main cause of environmental mastitis in Peruvian farms [51,52], animals were likely either treated unnecessarily (mild cases) or undertreated (critical cases requiring broader-spectrum antibiotics). Finally, given that ESBL-*E. coli* is also prevalent in humans from Peruvian rural areas, we cannot rule out the possibility that farmers themselves could be the source of strains for their animals, which could be assessed in the future by genome-based studies.

The prevalence of ESBL-*E. coli* was also high in pigs (61%), which could be explained by specific factors identified on pig farms. As observed in dairy cattle farms, inadequate implementation of biosecurity measures may increase the need for AMU. Macrolides were the primary choice for treating pigs with respiratory or gastroenteric diseases, which also differs from our previous observations in this region (i.e., primary use of penicillins for respiratory disorders and aminoglycosides for gastroenteritis [21]). The current AMU practices reported by farmers in this study resemble those reported in European countries [53], although, to our knowledge, there are no official guidelines implemented in the study region. However, pathogens’ prevalence may vary significantly across geographical regions, particularly between middle- and high-income countries, and there is no evidence that adopting European practices would improve animal health or reduce ESBL-*E. coli* in Peruvian livestock. For example, *E. coli* and *Salmonella* spp. were the most reported bacteria affecting pigs in Latin America between 2006 and 2016 [54]. While macrolides are effective against a broad range of Gram-positive and Gram-negative bacteria, *E. coli* and *Salmonella* spp. generally show low susceptibility to macrolides [53]. Thus, the frequent treatments with macrolides in pig herds in Peru, combined with a lack of laboratory diagnostics to confirm a bacterial origin, highlight potential antibiotic misuse in this species. In addition, the intense contact of livestock with dogs and rodents reported by farmers may be contributing to the emergence and maintenance of these antibiotic-resistant bacteria, as suggested by previous research in Brazil and Chile [7,17].

In contrast to previous research, we found a significantly lower prevalence of ESBL-*E. coli* in laying hens (34%) compared to pigs (61%) and dairy cows (75%). Pigs and poultry are often reported as the main sources of ESBL-*E. coli* on farms, attributed to different AMU patterns and specific husbandry practices [5,33,36,37,44]. However, we found a much lower prevalence in laying hens (34%) in Ica than the >70% previously reported by Dávalos et al. (2022) [23] in the same region. These differences may be linked to the type of poultry farming system studied. While our study focused on small-scale laying hen farms, the previous study was conducted in large-scale broiler production systems. Large farms typically use higher amounts of antibiotics, which can increase the selective pressure favoring the emergence and spread of ESBL-*E. coli* [12,55]. They also tend to have higher animal densities and higher numbers of staff, thereby increasing the risk of bacterial spread [55]. Differences in production systems may explain the lower ESBL-*E. coli* prevalence in laying hens. Broilers typically receive more antibiotics for growth and disease prevention, while laying hens are treated less frequently or with different drugs. Feed in broiler systems is often optimized to boost growth, potentially affecting gut microbiota and competition between antibiotic-resistant and susceptible bacteria. Additionally, broilers raised on litter face more heat stress and moist bedding, conditions that favor bacterial survival. In contrast, caged laying hens have limited contact with feces, reducing fecal–oral transmission of antibiotic-resistant bacteria. Fluoroquinolones for respiratory disorders and amoxicillin for gastroenteritis were the main antibiotics used in laying hens. These antibiotics are often used in egg-producing LMICs or neighboring countries to Peru such as Brazil, Argentina, Chile, and China [56,57,58,59]. In contrast, their use in laying hens is forbidden in Europe to avoid adverse effects on humans [60]. Several studies have demonstrated residues of fluoroquinolones and penicillins (i.e., amoxicillin) in eggs from treated chickens [61,62]. Although both diseases were rarely reported by farmers in our study area (0.34 respiratory occurrences/1000 animals and 0.13 gastroenteric occurrences/1000 animals in the last semester), individual treatments in poultry production are often inapplicable [10]. Thus, although our questionnaire did not include questions about the number of animals treated, unnecessary AMU in healthy chickens on our study farms was likely. AMU in laying hens raises public health concerns, as Peru has neither specific regulations for withdrawal periods nor maximum residue limits for antibiotics such as amoxicillin and fluoroquinolones in eggs [63,64]. Additionally, we confirmed these animals as carriers of ESBL-*E. coli,* even though these animals are often intended for human consumption at the end of their productive cycle [65,66].

Our study required a substantial effort to combine field sampling, questionnaire administration, and laboratory analyses to study the drivers of ESBL-*E. coli* among Peruvian farms. However, several limitations of our study could be addressed by future studies to deepen our understanding of this important question. First, our data on farmers’ characteristics and practices were obtained from farmers responses to a questionnaire without validation through systematic collection of observational data. This could introduce self-reporting bias and prevent a more quantitative assessment of several variables extracted from our questionnaire (e.g., frequency and percentage of animals affected by a given disease). Second, we did not collect detailed data on livestock antibiotic use, such as the amount used, duration, or routes of administration, which prevented the assessment of a more specific association with ESBL-*E. coli*. Likewise, clinical data on the proportion of animals treated, as well as animal-level data (e.g., age, history of disease and treatment), were not available, which precluded both estimating the rates of successful/unresponsive antibiotic treatments and assessing the correlations between ESBL-*E. coli* and disease occurrence and treatment at the animal level. Third, our statistical analyses (i.e., Poisson GLM) may have lacked sufficient statistical power to detect additional significant associations between ESBL-*E. coli* prevalence at the farm level and the 37 variables extracted from the questionnaires. Given the limited sample size and the large number of predictors, we employed a LASSO regression to objectively reduce the influence of less informative variables, thereby improving model generalizability. Although this conservative approach may have limited the detection of weaker associations, it helps avoid detecting statistically significant associations that result from poor model fitting and reduces the likelihood of variable multicollinearity affecting results [67,68]. This approach was complemented by an AIC-based selection approach on a limited number of selected variables that, although it introduced an additional bias into the selection of variables used in the model, yielded almost the same results (with the addition of one significant variable). We suggest that further research aiming to identify drivers of ESBL-*E. coli* should sample a much larger number of farms per animal species to obtain sufficient statistical power to detect all main significant drivers of the observed high ESBL-*E. coli* prevalence. This effort will require substantial funding to conduct the field work, questionnaire implementation, and laboratory work, as well as the logistical support of national public health authorities. A higher statistical power will also allow for testing non-linear effects of the studied variables, as well as using more complex model approaches such as generalized additive models or machine learning approaches. Finally, while phenotypic tests are highly sensitive and specific, future molecular analyses could reveal transmission pathways (shared resistance genes) or regional differences. Such analyses could also assess zoonotic potential through comparisons with available genomes of ESBL-*E. coli* isolated from humans and the environment.

## 4. Materials and Methods

### 4.1. Selected Study Regions

**Figure 6:**
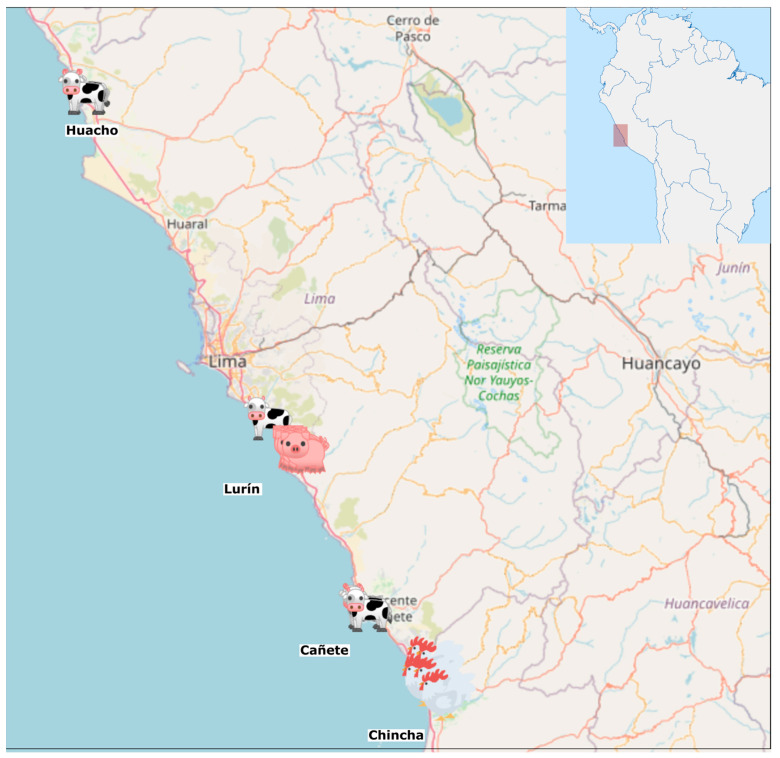
Study area in the Lima and Ica regions, Peru. The study area is highlighted by the red rectangle in the top right-hand corner map of Peru. Icons representing livestock indicate the approximate location of sampled farms. All silhouettes were obtained from creazilla.com and are available on an open-source license. The map was obtained from the open-sources websites OpenStreetMap (https://www.openstreetmap.org/) and Mapchart (https://www.mapchart.net/), accessed on 10 November 2024.

This study is part of a participatory research program aiming to co-construct, implement, and evaluate husbandry practices that reduce AMR circulation in partnership with small- and middle-scale farmers in the Lima (Huacho, Cañete, and Lurín districts) and Ica (Chincha district) regions in Peru (Figure 6). Lima has the largest livestock population of the country, with 72,321 dairy cows in production (8% of the country’s total) and more than 1.47 million pigs (40% of the country’s total) [24]. In 2023, its production exceeded 354 tons of milk and 200 tons of pig meat. We focused on dairy cows and pigs from farms in districts of Lima where we have previously estimated a high prevalence of ESBL-*E. coli* fecal carriage in these species (48%), potential wide antibiotic misuse, and poor farmers’ knowledge about antibiotics [6,21]. Ica is the largest region of laying hens and egg production in Peru, accounting for more than 10 million hens and 207 tons of eggs in 2023 [24,69], which is why this region was selected for the study of laying hens.

### 4.2. Livestock Sample Sizes and Farm Selection Design

Assuming a prevalence of 40% of fecal carriage in livestock based on our previous studies (48% prevalence of ESBL-*E. coli*; [6]), we estimated a sample size of 260 animals per livestock species to accurately estimate the prevalence with an error of 5%, a confidence level of 90%, and a population ranging between 100,000 to 1 million animals using the Epi info program [70]. The number of animals per farm in the studied areas varies significantly among dairy cattle, pig, and laying hen farms. Milk producers generally have fewer animals, with an average of 11 cows per farm in the Lima region [21], while laying hen farms in the Ica region may house thousands of chickens [23]. To detect at least one ESBL-*E. coli* isolate per farm with a probability of 90%, assuming a conservative 10% ESBL-*E. coli* prevalence per farm, we aimed to collect around 10 samples per dairy cattle and pig farm. Due to the higher number of animals, 25 samples were collected from each laying hen farm to ensure better representativeness. Thus, based on a total number of 260 animals per species, we aimed to sample 25 dairy cattle farms, 25 pig farms, and 10 laying hen farms.

Dairy cattle farms were identified by contacting the leadership of a local dairy cattle association (named *‘Asociación de Ganaderos de la Irrigación San Felipe’*) in the Vegueta district of Huaura of Lima. Likewise, two key pig professionals in the La Chutana district of San Bartolo of Lima (a veterinarian and a consultant) connected us with potential farmers. From a list of 100 farmers in the cattle associations and 50 identified pig farmers, we randomly selected the first 25 farms for each livestock species that met our inclusion criteria and asked them to participate. Finally, we identified 25 laying hen farms with the required characteristics from a list of farmers who had previously collaborated with either the Universidad Peruana Cayetano Heredia (Lima) or the Universidad Nacional San Luis Gonzaga (Ica). The laying hen farms were randomly selected until 10 farmers accepted participation in our study. We included all farms where the farmer voluntarily agreed to participate in the project. If a selected farmer declined the invitation, we randomly chose another farm from our list.

According to the Food And Agriculture Organization of the United Nations (FAO), there is no universally accepted definition of small-scale farms applicable to all countries, nor is there a national definition of the Peruvian small-scale livestock sector [71]. Classifications based on production characteristics are often used as they reflect structural constraints (e.g., limited access to land, resources, and/or technology) and associated low incomes [71,72]. For this study, we defined ‘small-scale’ farms as non-technified production systems lacking standardized or technological practices in feed management, egg collection, water quality control, waste management, and temperature/humidity regulation—criteria also used in previous studies in Peru [21,22,73].

This project was approved by the human and animal ethical committees (named *Comité Institucional de Ética en humanos* and *Comité Institucional de Ética para el uso de Animales*) of the Universidad Peruana Cayetano Heredia under the protocols 591-49-22 (human committee) and 050-12-22 (animal committee).

### 4.3. Fecal Sampling and Detection of E. coli Isolates with ESBL Phenotype

Between May and July of 2023, a total of 760 fecal samples were collected from 244 dairy cows (n = 25 farms), 261 pigs (n = 25 farms), and 255 laying hens (n = 10 farms). All samples were stored in Stuart transport medium and kept refrigerated (4–8 °C) until laboratory processing within 24 h [17]. Each sample was then streaked onto a MacConkey (Himedia^®^, Maharashtra, India) agar plate supplemented with 4 mg/L of cefotaxime to select potential ESBL-*E. coli* [74]. At least one isolate morphologically suspected of *E. coli* was submitted to species confirmation in chromogenic agar (HiCrome™️ ESBL Agar Base, Himedia^®^, Maharashtra, India), followed by biochemical tests to confirm them as *E. coli*, including the Sulfide Indole Motility, Simmons’ citrate, Urea, and Triple Sugar Iron [75]. Phenotypic ESBL production was assessed by the combination-disk test, according to Clinical and Laboratory Standards Institute (CLSI) guidelines [76]. Briefly, disks containing ceftazidime, ceftazidime/clavulanic acid, cefotaxime, and cefotaxime/clavulanic acid were applied on Müeller-Hinton (Himedia^®^, Maharashtra, India) agar plates. Isolates exhibiting an inhibition zone diameter at least 5 mm larger around the cephalosporins disks with clavulanic acid compared to the disks without it were considered positive for ESBL production. *Klebsiella pneumoniae* subsp. pneumoniae ATCC^®^ 700603™ was used as the positive control of ESBL production and *Escherichia coli* ATCC^®^ 25922™ (Microbiologics, St Cloud, MN, USA) was used as the negative control.

### 4.4. ESBL Genes Detection

To assess potential differences in the molecular mechanisms driving ESBL-*E. coli* across animal species or farm practices, we conducted a preliminary molecular analysis on a subset of randomly selected 78 ESBL-*E. coli* isolates (54 isolates from pigs, 18 from cows and 6 from hens). The presence of the most common genes of ESBL enzymes (*bla*_CTX-M_, *bla*_SHV_, and *bla*_TEM_) was tested using a multiplex PCR assay, as described in our previous study [17]. The *E. cloacae* NCTC 13464 containing the *bla*_CTX-M_ gene, the *K. pneumoniae* ATCC^®^ BAA-1705™ containing the *bla*_TEM_ gene, and the *K. pneumoniae* ATCC^®^ 700603™ containing *bla*_SHV_ gene were used as positive controls.

### 4.5. Questionnaires to Assess Farmers’ Socioeconomic Characteristics and Husbandry Practices

To identify farmers’ socioeconomic characteristics and husbandry practices associated with the fecal carriage of ESBL-*E. coli* among livestock, we analyzed a subset of a structured questionnaire administered in person to the selected farmers. The complete questionnaire, adapted from our previous studies [17,21], is part of a larger project aiming to estimate the risks of interspecies transmission of AMR in enterobacteria among livestock, companion animals, humans, and the environment. Questions relevant to this study’s objectives were selected through discussions within our multidisciplinary research team including veterinarians, epidemiologists, and anthropologists (see Supplementary Material S1). We analyzed 17 questions that were distributed across four sections: (I) Farmer’ socioeconomic status (n = 4 questions), (II) farm’s characteristics and husbandry practices (n = 4 questions), (III) incidence and management of animal diseases (n = 3 questions), and (IV) biosecurity practices (n = 6 questions). AMU in livestock was assessed by asking farmers for details on treatments carried out in the last six and 12 months on their animals, focusing on the names of the products administered. AMU was recorded only when farmers reported either the brand-name or a specific active ingredient of an antibiotic used. To identify livestock AMU patterns, we categorized the antibiotics reported by farmers based on Magiorakos et al. [77] as follows: penicillins (i.e., penicillin and amoxicillin), cephalosporins (including third-generation cephalosporins), fluoroquinolones, phosphonic acids (fosfomycin), aminoglycosides, tetracyclines, macrolides, polypeptides (bacitracin), sulphonamides, and phenicols (florfenicol).

Questionnaires were administered to 59 adults (over 18 years old) in charge of each farm (referred here as ‘farmers’, the majority of whom were the farm’s owner). One farmer did not answer our questionnaire but allowed the sampling of their cows (n = 10 animals), so this farm was only included for the estimation of ESBL-*E. coli* fecal carriage prevalence in livestock.

### 4.6. Statistical Analyses

#### 4.6.1. ESBL-*E. coli* Prevalence Comparison Across Livestock Species

The prevalence of ESBL-*E. coli* per species and per farm was estimated as the number of animals (dairy cows, pigs, or laying hens) carrying at least one ESBL-*E. coli* isolate over the total number of animals sampled. Ninety-five percent confidence intervals were calculated using the function *binom.confint* (Agresti–Coull method) of the package *binom* in the R 4.2.1 software [42]. Statistical differences in prevalence between species were tested using the Chi-square test in R, using the Bonferroni correction method to control for the overall type I error rate [78,79].

#### 4.6.2. Association of ESBL-*E. coli* with Socioeconomic Characteristics and Husbandry Practices

**Figure 7:**
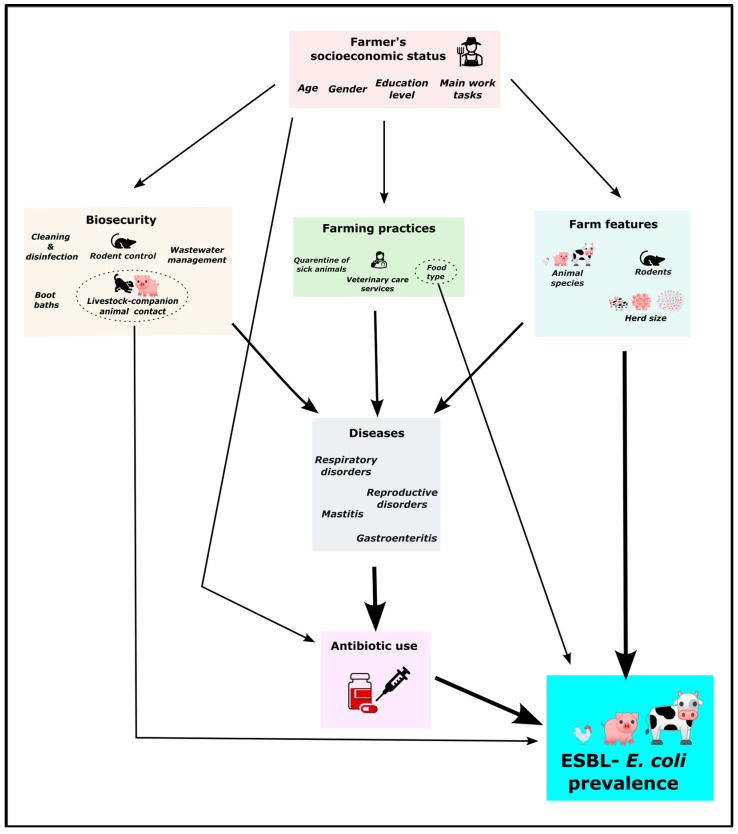
Conceptual diagram of the potential influence of the studied variables on ESBL-*E. coli* prevalence across livestock species in Peru.

To identify the main factors associated with the presence of animals carrying ESBL-*E. coli* on farms, we classified all obtained variables from the subset of selected questions into six categories: ‘Animal AMU’, ‘animal diseases’, ‘biosecurity’, ‘farm features’, ‘farming practices’, and ‘farmer’s socioeconomic status’ (Figure 7), extracting a total of 37 variables (see Supplementary Material S2). Then, we fitted a generalized linear model (GLM) with a Poisson distribution (Poisson GLM) using the number of ESBL-*E. coli*-positive animals per farm as the response variable. A Poisson regression model is well suited for count data. We also included an offset term for the logarithm of the total number of animals tested per farm to account for differences in sampling effort. This offset ‘log of the number of sampled animals’ adjusts the expected *ESBL*-*E. coli* count according to how many animals were sampled without estimating an additional parameter, ensuring comparability across farms. We tested the model’s overdispersion, outliers, and proper fitting using the *DHARMa* package in R. The lack of overdispersion assessed precluded the necessity of using other more complex alternative models accounting for overdispersion in our analyses (e.g., negative binomial model). Given limited statistical power due to the relatively small sample size (initially 59 farms), which prevented testing all 37 explanatory variables, we used the LASSO (Least Absolute Shrinkage and Selection Operator) method to perform automatic and penalized variable selection [80]. LASSO is particularly appropriate when the number of candidate predictors is large relative to the number of observations [81,82]. All available variables were included in the LASSO procedure without pre-filtering, allowing the model to select among all potentially relevant predictors (see Supplementary Material S2). However, due to missing values and categories represented by only a single observation, several rows had to be excluded, reducing the final number of usable observations to 49.

Acknowledging that this conservative approach could reduce the power to detect statistically significant correlates of ESBL-*E. coli*, we also performed an AIC-based model selection on two additional models, including (i) all 37 variables and (ii) 13 variables selected from the 37 variables. The 13 variables included 1–2 variables from each of the five variable categories (i.e., antibiotic use (n = 1), biosecurity (n = 2), animal diseases (n = 2), farm features including the animal species kept (n = 2), farming practices (n = 2)), as well as all four variables of farmer characteristics (Table 1). These variables were selected in each category according to several criteria, such as their occurrence in all animal species studied (e.g., exclusion of mastitis since it does not affect hens), their previous evidence of impact on the literature [7,17,83,84], and their highest variance among highly correlated variables within the same category (e.g., variable ‘antibiotic use in the last 12 months’ selected over the variable ‘antibiotic use in the last 6 months’). The AIC selection of the model that included all 37 variables resulted in poor model fit (e.g., overdispersion) and overfitting, and is therefore not presented in the results. The statistical significance of each variable included in our model, selected by either the Lasso or the AIC procedure, was then assessed by the Wald’s test, considering a significant effect with a *p*-value < 0.05. All explanatory variables submitted to the LASSO and AIC method for selection are available as Supplementary Material S2.

## 5. Conclusions

This study confirms the wide occurrence (98% of farms) and high prevalence of ESBL-*E. coli* fecal carriage among dairy cows and pigs in the Lima region of Peru, previously found in small-scale multi-species farms, while prevalence in laying hens in the Ica region was less than half. Although several antibiotics were used by farmers, antibiotic use did not correlate with the prevalence of ESBL-*E. coli* at the farm-level. Instead, only a farmer’s involvement in both the farm’s husbandry and animal healthcare, as well as a low number of gastrointestinal outbreaks on the farm, were associated with a lower prevalence of ESBL-*E. coli* at the farm. Overall, our study contributes to the limited data on AMR risk factors across multiple livestock species in Latin America and encourages future research with larger sample sizes and experimental designs (e.g., collecting data on antibiotic use and farming practices beyond self-reporting) to identify the main drivers of AMR. In particular, co-constructed interventions specifically reducing the risk factors found in this and other studies (e.g., gastrointestinal infections, specific characteristics of cattle farms) would allow for evaluating if those factors are responsible for the high prevalence of ESBL-*E. coli* found in our study area. Beyond their zoonotic potential [85,86], ESBL-*E. coli* can negatively impact farm productivity (e.g., reducing milk production due to mastitis) and harm the economic revenue of low-income farmers [43,87]. Therefore, our findings provide a starting point for identifying locally adapted strategies to limit the burden of ESBL-*E. coli*, safeguarding public health and fostering the economic sustainability of the Peruvian livestock sector.

## Figures and Tables

**Table 1:** Summary of farmers’ characteristics obtained from 59 questionnaires.

Category	Variable	Number of Farmers (*n*) (Total *n* = 59)	%
Gender	Women	26	44%
	Men	33	56%
Age	18–30	5	8%
	31–40	15	25%
	41–50	12	20%
	51–65	21	36%
	Over 65 years old	6	10%
Education level	None	2	3%
	Elementary	17	29%
	Secondary	26	44%
	Higher education	14	24%
Main work tasks	Administrative	17	29%
	Animal healthcare	2	3%
	Animal husbandry	24	41%
	Animal healthcare and husbandry	16	27%

## Data Availability

The data presented in this study are available within this article or as Supplementary Material.

## Supplementary Materials

The following supporting information can be downloaded at: https://www.mdpi.com/article/10.3390/antibiotics14090867/s1, S1: Questionnaires, S2: Explanatory variables used in the statistical analyses.
