# Supplementary material for: Unexplained High Prevalence of ESBL-Escherichia coli Among Cattle and Pigs in Peru
Source: Antibiotics (Basel). 2025 Aug 28;14(9):867. doi: 10.3390/antibiotics14090867 (PMC12466458; doi:10.3390/antibiotics14090867)
Supplement: Supplementary file 1 [file antibiotics-14-00867-s001.zip › antibiotics-3670631_Supplementary_Material_S1.pdf]

*(English version below)*

**Fecha_________**
**Nombre del centro de producción animal (CPA) ____________________________**
**Distrito y Provincia: __________________**
**Coordenadas________________________**

## CONDICIÓN SOCIOECONOMICA DE LOS GRANJEROS

**1. Sexo:** ( ) hombre ( ) mujer

**2. Edad en años:** ( ) 18-30 ( ) 31-40 ( ) 41-50 ( ) 51-65 ( ) mayor a 65

**3. Grado de instrucción: ____________________________________**

**4. ¿Qué tipo de trabajo realiza en el CPA? ________________________**

## CARACTERISTICAS DE LAS GRANJAS Y PRACTICAS DE MANEJO

**1. Su abastecimiento de agua es:** ( ) pozo ( ) canal de regadío/acequia ( ) red pública ( ) rio.

**2. Características de los animales criados**
Especie animal:
Propósito de crianza:
Cantidad de animales en la finca:

**3. Tipo de alimentación proporcionada a los animales:** ( ) Alimento balanceado ( ) Residuos de alimentos humanos ( ) Otros, especificar

**4. ¿Existen roedores en su CPA?** ( ) Si ( ) No

## INCIDENCIA Y MANEJO DE ENFERMEDADES EN LOS ANIMALES

**1. Enfermedades e tratamientos en el plantel en el último semestre**

| Enfermedad | # episodios sin tratar | # episodios tratados | Nombre del producto con el que los trato | ¿Funciono el tratamiento? |
|---|---|---|---|---|
| Respiratoria | | | | |
| Gastrointestinal | | | | |
| Reproductiva | | | | |
| Mastitis | | | | |

**2. ¿Aísla a los animales enfermos?** ( ) Si ( ) No

**3. ¿Qué servicios de sanidad animal utiliza para el ganado?**
( ) SENASA/Sector público
( ) Privado
( ) Tienda agropecuaria
( ) Ninguno
( ) Otro

## PRÁCTICAS DE BIOSEGURIDAD

**1. ¿Realiza algún tipo de control de roedores?** ( ) Si ( ) No

**2. ¿Cómo gestiona las aguas residuales en su CPA?**

**3. ¿Realiza con frecuencia limpieza y desinfección de las instalaciones?**
( ) Sí, ¿cuál es la frecuencia? ______________________ ( ) No

**4. Liste los principales productos usados en limpieza y desinfección:**

**5. ¿Posee su CPA pediluvios/rodaluvios para el ingreso de personas y/o vehículos?** Si ( ) No ( )

**6. Con qué frecuencia cambia los pediluvios?** ( ) Diaria, () Semanal () Mensual () Anual ()Nunca

**7. ¿Los perros tienen contacto cercano a los animales de producción?** ( ) Sí ( ) No

**ENGLISH VERSION……………………………………………..……………………………………………**

**Date_________**
**Name of the animal production center (APC) ____________________________**
**District and Province __________________**
**Coordinates________________________**

## FARMERS' SOCIOECONOMIC STATUS

**1. Sex:** ( ) male ( ) female

**2. Age in years:** ( ) 18-30 ( ) 31-40 ( ) 41-50 ( ) 51-65 ( ) more than 65

**3. Education level: ____________________________________**

**4. What type of work do you perform at the APC? ________________________**

## FARM CHARACTERISTICS AND MANAGEMENT PRACTICES

**1. Your water supply is:** ( ) well ( ) irrigation canal/ditch ( ) public network ( ) river

**2. Characteristics of the animals raised**

Animal species:

Purpose of raising:

Number of animals on the farm:

**3. Type of feed provided to the animals:** ( ) Balanced feed ( ) Human food waste ( ) Other, specify: ___________

**4. Are there rodents on your farm?** ( ) Yes ( ) No

## INCIDENCE AND MANAGEMENT OF ANIMAL DISEASES

**1. Diseases and treatments in the herd in the last semester**

| Disease | # untreated episodes | # treated episodes | Name of the product used | Did the treatment work? |
|---|---|---|---|---|
| Respiratory | | | | |
| Gastrointestinal | | | | |
| Reproductive | | | | |
| Mastitis | | | | |

**2. Do you isolate sick animals?** ( ) Yes ( ) No

**3. What animal health services do you use for your livestock?**

( ) SENASA/Public sector
( ) Private
( ) Agricultural supply store
( ) None
( ) Other: _______________

## BIOSECURITY PRACTICES

**1. Do you perform any type of rodent control?** ( ) Yes ( ) No
**2. How do you manage wastewater on your farm?**
**3. Do you regularly clean and disinfect the facilities?** ( ) Yes, how often? __________________ ( ) No

**4. List the main products used for cleaning and disinfection:**

**5. Does your farm have footbaths/wheel baths for the entry of people and/or vehicles?** ( ) Yes ( ) No

**6. How frequently you change footbaths?** ( ) Daily, () Weekly () Monthly () Annually ()Never

**7. Do dogs have close contact with production animals?** ( ) Yes ( ) No
